# Why Some Employees Adopt or Resist Reorganization of Work Practices in Health Care: Associations between Perceived Loss of Resources, Burnout, and Attitudes to Change

**DOI:** 10.3390/ijerph110100187

**Published:** 2013-12-20

**Authors:** Carl-Ardy Dubois, Kathleen Bentein, Jamal Ben Mansour, Frédéric Gilbert, Jean-Luc Bédard

**Affiliations:** 1 Faculty of Nursing, University of Montreal, C.P. 6128, succ. Centre-ville, Montréal, QC H3C 3J7, Canada; 2 École des Sciences de la Gestion, Université du Québec à Montréal, 315, Rue Sainte-Catherine Est, Montréal, QC H2X 3X2, Canada; E-Mails: bentein.kathleen@uqam.ca (K.B.); gilbert.frederic@uqam.ca (F.G.); 3 Département des Sciences de la Gestion, Université du Québec à Trois-Rivières, 3351, Boul. des Forges, C.P. 500, Trois-Rivières, QC G9A 5H7, Canada; E-Mail: Jamal.Ben.Mansour@uqtr.ca; 4 Institut National de la Recherche Scientifique, Centre Urbanisation Culture Société, 385, Rue Sherbrooke Est, Montréal, QC H2X 1E3, Canada; E-Mail: jean-Luc.Bedard@ucs.inrs.ca

**Keywords:** work organization, burnout, change management, conservation of resources theory

## Abstract

In recent years, successive work reorganization initiatives have been implemented in many healthcare settings. The failure of many of these change efforts has often been attributed in the prominent management discourse to change resistance. Few studies have paid attention to the *temporal process* of workers’ resource depletion/accumulation over time and its links with workers’ psychological states and reactions to change. Drawing upon the conservation of resources theory, this study examines associations between workers’ perceptions of loss of resources, burnout, and attitudes to change. The study was conducted in five health and social service centres in Quebec, in units where a work reorganization project was initiated. A prospective longitudinal design was used to assess workers’ perceptions at two time points 12 months apart. Our findings are consistent with the conservation of resources theory. The analysis of latent differences scores between times 1 and 2 showed that the perceived loss of resources was associated with emotional exhaustion, which, in turn, was negatively correlated with commitment to change and positively correlated with cynicism. In confirming the temporal relationship between perceived loss of resources, occupational burnout, and attitude to change, this research offers a new perspective to explain negative and positive reactions to change implementation.

## 1. Introduction

In recent years, successive work reorganization initiatives have been implemented in many healthcare settings to improve work processes, increase efficacy and efficiency, and respond to external pressures (e.g., budgetary constraints, human resource shortages, consumer calls for improved care quality). 

Despite claims of these initiatives’ potential benefits for both employers and workers, organizations often experience unforeseen difficulties and setbacks in implementing them. Studies reveal that, in many cases, these new work practices have been used only in a piecemeal and partial way due to implementation difficulties [1,2,3,4]. The failure of many of these change efforts has often been attributed in the prominent management discourse to change resistance [5]. There have been suggestions that employees’ attitudes toward change are a key factor in determining the success of these work reorganizations [6]. However, more research is needed to understand better the development of these attitudes. Until now, little work has been undertaken to examine systematically why many organizations have failed to engage their workers in introducing new forms of work organization. The limitations of resistance to change as the sole explanation have been underlined [6,7,8]. Some analysts suggest that greater attention should be paid to the psychological processes underlying this resistance and their consequences. While workplace innovation can have positive outcomes for employees, there is also evidence of a downside to new forms of work organization, which can result in psychological manifestations such as stress, burnout, and other disruptions that may be considered antecedents to change resistance. In conceptualizing the psychological process of “resistance”, some researchers refer to feelings or threat of loss experienced by people in a context of change. Dent and Goldberg [7] argue that people do not resist change *per se*, but may resist loss of status, loss of pay, or loss of comfort. In the same vein, van Dijck and van Dick [9] suggest that what is conceptualized as resistance to change is actually an employee response to a threat to work-based identity, linked to a perception of loss of group status. In the present paper, we examine the development of employee attitudes toward change as the result of a reaction to feelings of loss. We postulated that perceptions of losses associated with changes in the workplace and the resulting psychological manifestations such as burnout may undermine workers’ commitment to change and generate cynicism toward change management.

### 1.1. Study Objective

The aim of the present research was to explore the relationships between resource loss over time, burnout, and attitudes toward change (commitment to change and cynicism toward change management). 

### 1.2. Theoretical Background

The research reported here builds mainly on the conservation of resources (COR) theory. This theory offers a useful framework to understand the relationships between the resource loss that characterizes an organizational change and employees’ attitudes toward that change. Central to this theory is the assumption that the prime human motivation is directed toward resource maintenance and accumulation [10,11,12]. Resources are defined as those objects (e.g., tools), personal characteristics (e.g., emotional stability), conditions (e.g., social support), or energies (e.g., money) that are either valued in their own right by the individual or that serve as a means to acquire or protect other valued resources [10]. Given the objective of this study, our focus was on job resources (*i.e.*, conditions). Drawing on conceptualizations from previous studies [13,14,15] and based on the specific nature of work in healthcare, we considered job resources located at four levels: task (autonomy, and opportunities for stimulating work), interpersonal (group cohesion), supervision (supervisor support), and organizational (informal power). 

According to the COR theory [11,12], people strive to retain, protect, and build those different types of resources. Psychological manifestations such as burnout will occur when people experience the potential or actual loss of these valued resources or fail to gain sufficient resources after significant resource investment. Burnout is an individual’s reaction to an environment in which there is either a threat or actual loss of resources, or lack of an expected gain in resources. The initial threat to resources could be seen as a stressor, and the continued loss or threat to resources over time, particularly after a great deal of resource investment in work, is said to lead to burnout. Recent empirical studies support the COR theory, and particularly the association between resource depletion and burnout [16,17]. The COR theory also suggests that when burned-out employees experience a significant depletion of resources, they adopt a defensive strategy for investing their remaining resources [11,18]. Several studies have provided empirical support to this process. It has been shown that employees who experience resource loss or threat of loss tend to protect their remaining resources and develop new strategies to maximize the return associated with their resource investments [19,20].

### 1.3. Hypotheses

Based on the theoretical framework and suggestions from the empirical literature, we tested three hypotheses:

**Hypotheses** **1a**, **1b**, **1c**, **1d**, **1e**, that over the study period, resource loss (H1a_[autonomy]_, H1b_[opportunities for stimulating work]_, H1c_[group cohesion]_, H1d_[supervisor support]_, H1e_[informal power]_) would be positively related to increase in burnout.

**Hypothesis** **2**, that over the study period, increase in burnout would be negatively related to commitment to change.

**Hypothesis** **3**, that over the study period, increase in burnout would be positively related to cynicism toward change management.

## 2. Methods

### 2.1. Sampling

The study was carried out in five health and social service centres in Quebec where explicit work reorganization projects were initiated. In each centre, field work for this study was restricted to the units where the selected work reorganization project was implemented. Project selection was guided by three criteria: scope of the project (work reorganization initiative involving a critical mass of workers); its innovative nature (clear differences with previous work practices); and its degree of formalization (explicit objectives, resources, and strategies). In all five settings, the work reorganization projects were intended to address human resource shortages by introducing new work practices that fostered better use of healthcare workers. In three projects, the main strategies used were the introduction of support workers to complement professional work, the revision of roles, and the creation of conditions to improve collaboration. In the two other projects, the main strategies used were the introduction of new forms of supervision and the revision of work processes.

Based on data provided by managers at each site, we determined that a total of 389 employees were involved in the work reorganization projects at the time of the baseline survey. This number remained stable at 386 at the time of follow-up 12 months later. The study included all employees who had worked actively, either part-time or full time, as care providers for at least three months in one of the units where the selected work reorganization projects were introduced. The final sample consisted of a diverse mix of employees that included registered nurses, assistant nurses, rehabilitation workers (occupational therapists and rehabilitation technicians), and psychosocial workers (psychologists, social workers). As the study’s focus was on frontline staff involved in direct patient care, managers, administrative staff, and clerical workers were not included.

### 2.2. Data Collection

A questionnaire was sent, at two time points 12 months apart, to all employees who met the inclusion criteria. At baseline, 254 workers (65%) completed and returned the questionnaire, and 202 (51%) responded to the follow-up 12 months later. The baseline coincided with the preparation phase of the work reorganization projects (communication about the project, advocacy, implementation planning). The second wave of data collection took place when the implementation phase was well advanced and workers had experienced the new work practices. This paper is based on data from 96 participants for whom measures were taken at both time points. To determine whether attrition produced difference in the usable sample, we used dummy variables to classify respondents into two groups: Group 1 (G_1_), those who completed measures only at T1 (N_1_ = 155), and Group 2 (G_2_), those who completed measures at both T1 and T2 (N_2_ = 96). We examined whether the two groups differed on gender, age, occupation, and job status. The omnibus F ANOVA test indicated no differences between the two groups on any of those variables (F_[gender]_ = 1.50, ρ = 0.22; F_[age]_ = 1.92, ρ = 0.16; F_[occupation]_ = 0.28, ρ = 0.59; F_[status]_ = 0.84, ρ = 0.35). We concluded that these 96 respondents did not differ from the others with respect their characteristics (gender, age, occupation, job status). In summary, attrition from T1 to T2 did not appear to create a bias with regard to the demographic variables.

### 2.3. Variables and Instruments

#### 2.3.1. Job Resources

Job resources were measured using five indicators widely used in organizational research and that cover four levels of work organization: autonomy and opportunities for stimulating work at the task level; group cohesion at the interpersonal level; supervisor support at the supervision level; and informal power at the organizational level. These indicators connect to critical resources (control over one’s work, social support, rewards from working) whose depletion has been associated by Maslach *et al.* [21] with development of burnout. Examples of items used to operationalize each of these five indicators are presented in Table 1.

**Table 1:** Examples of instruments’ items.

Construct	Examples of Items
Autonomy	“I have almost complete responsibility for deciding how and when the work is to be done.”
Opportunities for stimulating work	“How much do you have in your present job the chance to gain new skills and knowledge on the job.”
Informal power	“Collaborating on patient care with physicians; being sought out by peers for help with problems.”
Group cohesion	“The members of my work group stand up for each other.”
Supervisor support	“My supervisor takes pride in my accomplishments at work.”
Burnout	“I feel emotionally drained from my work.”
Commitment to change	“I am doing whatever I can to help this change be successful.”
Cynicism	“I believe that management motives for this change are different from those stated publicly.”

Autonomy was assessed using three items from the Job Descriptive Scale [22]. The three items were rated on a 7-point Likert scale ranging from 1 (strongly disagree) to 7 (strongly agree).

Respondents’ perceptions of access to opportunities for stimulating work and informal power were measured with the corresponding subscales of the Conditions of Work Effectiveness Questionnaire-II [23]. The scale to assess “opportunities for stimulating work” comprised three items and the scale for “informal power” had four. Respondents were asked to rate each item in terms of how often it happened in their job, on a scale from 1 (never) to 5 (very often). For example, to measure access to opportunity, respondents were asked to rate the degree to which their job gave them the opportunity to perform challenging work or use their knowledge and skills.

Group cohesion was measured with the three-item scale from Podsakoff and MacKenzie [24], and supervisor support with the six items used by Stinglhamber and Vandenberghe [25], a French version of the Eisenberger scale [26]. In both cases, items were rated on a 5-point scale ranging from 1 (strongly agree) to 5 (strongly disagree).

#### 2.3.2. Burnout

Burnout was measured in this study using the nine-item Emotional Exhaustion subscale of the Maslach Burnout Inventory (MBI) [21]. Although Maslach’s original conceptualization of burnout [27,28] includes three components (emotional exhaustion, depersonalization, and diminished personal accomplishment), the nine-item Emotional Exhaustion subscale is the most widely used in workforce research, and there is growing consensus in the recent literature that emotional exhaustion is conceptually the “core meaning” of burnout [29,30,31]. Some researchers have developed and empirically supported a process model of burnout in which emotional exhaustion plays a central role in predicting the two other components of burnout: depersonalization and diminished personal accomplishment [32,33]. Related to this, other works have shown that emotional exhaustion exhibits stronger relationships to important outcome variables than do the two other components [14,30,34]. 

Emotional exhaustion refers to feelings of being emotionally overextended and exhausted by one’s work, and manifests as both physical fatigue and a sense of feeling psychologically and emotionally “drained”. Respondents were asked to rate the frequency with which they experienced the feelings referred to in each item on a 7-point Likert-type scale ranging from 0 (never) to 6 (daily).

#### 2.3.3. Attitudes to Change

Attitudes to change were operationalized in this study by two constructs: commitment to change and cynicism toward change management. Commitment to change measures an actual or future behavioural intent that results from employees’ attachment to a change initiative and their willingness to contribute to its success. It was assessed with a 3-item scale from Fedor, Caldwell, and Herold [35]. Cynicism measures employees’ beliefs of unfairness and feelings of distrust toward change management; it was captured by an 8-item scale from Stanley, Meyer, and Topolnytsky [36]. 

### 2.4. Analysis Techniques

Analyses were conducted using structural equation modeling (SEM) with Mplus 6.12 software package. To test our hypotheses, we introduced a latent difference score (LDS) [37,38]. Compared to classical methods (e.g., difference scores, gain scores, repeated-measures ANOVA), the LDS approach does not suffer from issues associated with measurement error. It is maximally reliable and therefore less likely to introduce biases into the hypothetical models to be tested. LDS is particularly useful to model mean change over time as well as individual differences around that mean change. For each construct used in this study, we introduced a latent construct (∆f) that represented the latent change (growth or decline) between two common-factor scores (f[T1] and f[T2]) for this construct measured at two times (e.g., ∆autonomy[T2−T1]; ∆burnout[T2−T1]). The variance represents interindividual differences in intraindividual change from T1 to time T2 on the construct (f). A negative mean score indicates a true decline in the construct for an individual, whereas a positive mean score indicates a true increase and a score of zero indicates no true change at all.

**Figure 1:**
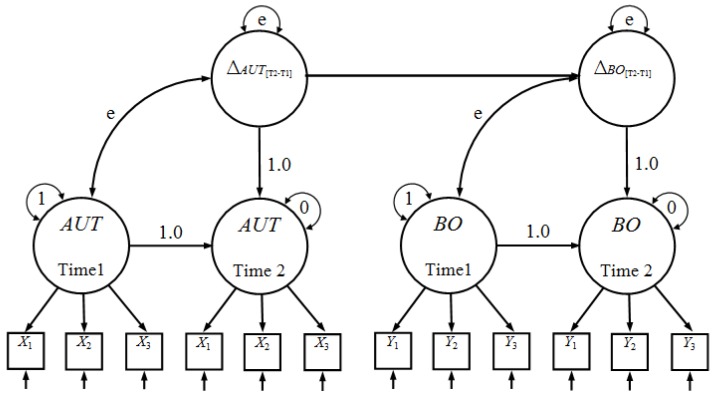
A model using a path graphic representation with two factors.

Because this study focused on the dynamic interplay among several changing constructs that evolved over time, the LDS model was extended to a multiple-LDS model with paths linking the latent differences to each other. This extension of latent difference scores can be used to test hypotheses that changes in resources are a primary predictor of subsequent changes in burnout between time 1 and time 2. Thus, a multiple-common-factor latent change model (a model combining two or more latent change constructs ∆f, ∆g, ...) and its parameters (variance, covariance, and path coefficients) allowed us to test hypotheses about change over time related to two or more constructs, e.g., the relationship between latent change in autonomy (∆autonomy[T2−T1]) and latent change in emotional exhaustion (∆emotional-exhaustion[T2−T1]). Figure 1 portrays such a model using a path graphic representation with two factors: autonomy (AUT) and burnout (BO). 

## 3. Results

### 3.1. Descriptive Statistics

Table 2 shows the means, standard deviations, intercorrelations between variables, and internal consistencies reliability (Cronbach’s alpha) of the different measurement scales used in this study. Except for informal power (Cronbach’s alpha of .69 at T1 and T2), all scales showed relatively high reliability, ranging from 0.79 to 0.95. The reliabilities of a same scale remained stable across time. The zero-order correlations did not indicate any problem of multicollinearity.

Of the 96 respondents, 12.5% were men and 87.5% were women; 68.8% were permanent full-time employees, 20.8% were permanent part-time, and 10.4% were employed on a temporary basis. The average number of hours worked per week was 33.48, and 96.8% of respondents worked on the day shift. The average occupational tenure was 9.54 years (SD = 7.14), and the average organizational tenure was 9.6 years (SD = 7.7). 

**Table 2:** Descriptive statistics and correlations among the study variables.

	M	SD	1	2	3	4	5	6	7	8	9	10	11	12	13	14
**1. OPP-T1**	4.00	0.68	(0.79)													
**2. OPP-T2**	3.98	0.66	0.37 ***	(0.80)												
**3. IP-T1**	3.38	0.66	0.16	0.14	(0.69)											
**4. IP-T2**	3.24	0.77	0.17	0.36 ***	0.66 ***	(0.69)										
**5. GC-T1**	3.75	0.70	0.07	0.10	0.22 *	0.12	(0.81)									
**6. GC-T2**	3.59	0.93	0.13	0.26 *	0.25 *	0.32 **	0.55 ***	(0.89)								
**7. SS-T1**	3.63	0.67	0.39 ***	0.30 **	0.41 ***	0.38 ***	0.31 **	0.31 **	(0.89)							
**8. SS-T2**	3.64	0.78	0.26 *	0.44 ***	0.19	0.39 ***	0.11	0.35 ***	0.49 ***	(0.92)						
**9. AUT-T1**	5.27	1.00	0.27 *	0.39 ***	0.17	0.21 *	0.18	0.40 ***	0.40 ***	0.52 ***	(0.84)					
**10. AUT-T2**	5.04	1.19	0.26 *	0.50 ***	−0.05	0.31 **	0.11	0.45 ***	0.28 *	0.40 ***	0.50 ***	(0.86)				
**11. BURNOUT-T1**	2.11	0.98	−0.06	−0.06	0.01	−0.02	0.02	0.04	−0.07	−0.08	−0.18	−0.13	(0.87)			
**12. BURNOUT-T2**	2.12	1.09	−0.09	−0.29 **	0.15	−0.22 *	−0.06	−0.25 *	−0.22 *	−0.26 **	−0.23 **	−0.39 ***	0.58 ***	(0.89)		
**13. CC-T2**	3.56	0.64	0.00	0.29 **	0.37 ***	0.50 ***	−0.03	0.32 **	0.45 ***	0.44 ***	0.32 ***	0.32 **	−0.12	−0.28 *	(0.82)	
**14. CYN-T2**	2.83	0.86	−0.13	−0.40 ***	−0.29 **	−0.37 ***	−0.11	−0.49 ***	−0.43 ***	−0.34 ***	−0.35 ***	−0.50 ***	0.15	0.42 ***	−0.60 ***	(0.95)

Notes: N = 96; Alpha coefficients are reported diagonally; OPP = opportunity; IP = informal power; GC = group cohesion; SS = supervisor support; AUT = autonomy; CC = commitment to change; CYN = cynicism; * *p* < 0.05; ** *p* < 0.01; *** *p* < 0.001.

### 3.2. Confirmatory Factor Analyses

Before testing our hypotheses, we evaluated the discriminant and convergent properties of our measures with confirmatory factor analysis (CFA) via a sequence of nested models [39], presented in Table 3. We compared the fit of two nested models: an eight-factor model and a seven-factor alternative model. The eight-factor model (Mod-F_8_) was obtained by loading items on their respective scales: autonomy, opportunities for stimulating work, group cohesion, supervisor support, informal power, burnout, commitment to change, and cynicism. The seven-factor model (Mod-F_7CC + CYN_) was obtained by combining variables related to attitude to change (commitment to change and cynicism). Table 3 displays fit indices for the measurement models. As can be seen, the chi-square difference tests indicated that the constrained seven-factor model (Mod-F_7CC + CYN_) displayed significant decrements in fit as compared with the eight-factor model (Mod-F_8_). Consequently, we used the eight-factor model to examine hypotheses 1–3.

**Table 3:** Confirmatory factor analysis (CFA).

**Model (Time 1)**	**χ^2^**	**df**	**CFI**	**RMSEA**	**SRMR**
**Mod-F_8_: Eight factors**	**681.34**	**467**	**0.90**	**0.07**	**0.06**
Mod-F_7CC+CYN_ : seven factors	772.32	474	0.84	0.08	0.08
∆χ^2^ (Mod-F_8_ *vs*. Mod-F_7CC + CYN_)	90.98 ***	7	––	––	––
**Model (Time 2)**	**χ^2^**	**df**	**CFI**	**RMSEA**	**SRMR**
**Mod-F_8_: Eight factors**	**733.30**	**467**	**0.89**	**0.07**	**0.07**
Mod-F_7CC + CYN_: seven factors	822.88	474	0.85	0.08	0.08
∆χ^2^ (Mod-F_8_ *vs*. Mod-F_7CC + CYN_)	89.58 ***	7	––	––	––

Notes: df = degrees of freedom; CFI = comparative fit index; RMSEA = root-mean-square error of approximation; SRMR = standardized root mean square residual; ∆χ^2^ = chi-square difference tests between the eight-factor model and alternative models; * *p* < 0.05; ** *p* < 0.01; *** *p* < 0.001.

### 3.3. Univariate Models

In this section, we describe results from univariate LDS models. First, as a preliminary step, we examined the longitudinal measurement invariance (configural and metric) to confirm that construct meaning items on a particular instrument assessed the same attribute with the same degree across time. The results show overall strict measurement invariance for all instruments, indicating good measurement consistency over time. For example, autonomy provided a good fit to the data for the configural invariance test between T1 and T2 (χ^2^ = 8.67; df = 5; N = 96; CFI = 0.98; RMSEA = 0.08; SRMR = 0.04) and for the metric invariance test between T1 and T2 (χ^2^ = 11.54; df = 7; N = 96; CFI = 0.98; RMSEA = 0.08; SRMR = 0.06).

Second, we examined change in both resources’ indicators and burnout from T1 to T2. LDS analyses showed that the means of latent difference factors were significantly different from 0 for all resources (μ(∆f) ≠ 0). Except for supervisor support, the trajectories of all other resources decreased over time (μ(∆f_[T2−T1]_) < 0) (μ_[autonomy]_ = −0.18 ***; μ_[opportunities for stimulating work]_ = −0.03 ***; μ_[group cohesion]_ = −0.21 ***; μ_[informal power]_ = −0.18 ***). The trajectory for supervisor support increased over time (μ_[supervisor support]_ = 0.02 ***). This means that during the period of the study (over 12 months), respondents perceived a gain in supervisor support and losses in the other four resources. With regard to burnout, the positive mean (μ_[burnout]_ = 0.06) of latent change difference indicated that, on average, individuals showed an increase over time. Importantly, all the latent difference factors had statistically significant variances (σ^2^_[autonomy]_ = 1.25; σ^2^_[opportunities for stimulating work]_ = 1.14; σ^2^_[group cohesion]_ = 1.37; σ^2^_[supervisor support]_ = 1.11; σ^2^_[informal power]_ = 0.42; σ^2^_[burnout]_ = 1.03).

### 3.4. Multivariate Models

To test our hypotheses, we used a model that included latent changes in resources and in burnout over 12 months and employee attitude toward organizational change (commitment to change and cynicism toward change management). The model provided an adequate fit to the data (RMSEA = 0.07; CFI = 0.94; SRMR = 0.07). With regard to hypothesis 1, our findings indicated that a decrease in resources was significantly associated with increase in burnout from T1 to T2 (β_[autonomy]_ = −0.36, ρ < 0.05; β_[opportunities for stimulating work]_ = −0.24, ρ < 0.05; β_[group cohesion]_ = −0.36, ρ < 0.05; β_[informal power]_ = −0.45, ρ < 0.000). However, no significant association was found between increase in supervisor support and increase in burnout (β_[supervisor support]_ = −0.13, ρ = 0.27). Hypotheses 1a, 1b, 1c and 1e were supported; hypothesis 1d was not supported. 

With regard to hypotheses 2 and 3, a significant positive relationship was found between increase in burnout and cynicism toward change management (β_[cynicism]_ = 0.37, ρ < 0.05), and a significant negative relationship was found between increase in burnout and commitment to change (β_[change commitment]_ = −0.27, ρ < 0.05). Hypotheses 2 and 3 were supported. 

As complementary analyses, we tested a model including a direct path from latent changes in resources (autonomy, opportunities for stimulating work, group cohesion, and informal power) to attitudes toward organizational change (commitment to change and cynicism toward change management). Results of these complementary analyses show that latent change in burnout completely mediated the relationship between latent changes in resources and attitudes toward organizational change, for all types of resources except group cohesion. For this last resource, the mediation by latent change in burnout was partial.

## 4. Discussion and Conclusions

To the best of our knowledge, this study makes a unique and original contribution by investigating a temporal and insufficiently explored relationship between perceived loss of resources, occupational burnout, and attitudes to change in the context of work reorganization in healthcare. Although work reorganization projects are often promoted by top corporate and managerial staff with promises of better work conditions for employees, a first finding of this study is that the immediate change process resulting from these initiatives often elicits less optimism than expected from frontline staff. Indeed, this study showed that the implementation of these changes was associated with perceptions of loss of diverse resources among frontline staff, as reflected in the observed decrease over time in perceived autonomy, perceived opportunities for stimulating work (task level), perceived cohesion (interpersonal level), and perceived informal power (organizational level). Among the set of resources studied, only perceived supervisor support (supervision level) increased over time for frontline staff involved in the selected work reorganization projects, and this could be due to the prominent role often delegated to middle managers in the implementation of these changes. These results are consistent with findings from earlier studies showing that, in many cases, work reorganization initiatives alter work environments in ways that expose workers to a potent mix of resource constraints, heavy workloads, work standardization, and added responsibilities, without the necessary reciprocal support [40,41,42]. In our study, although resources related to support from the immediate supervisor increased over time, other forms of resources related to team and organizational support decreased. While previous studies in this domain have often tended to focus on job demands to explain workers’ reactions to new forms of work organization, the present research highlights the central role of personal resources in these processes.

Consistent with hypothesis 1, this study revealed a positive association between a decrease in resources over time and an increase in burnout over that same time. This result confirms the contention of the COR theory [10] that employees’ perceptions of loss of valued resources in a context of change may impact on their psychological state and result in burnout. This result also echoes findings from several studies suggesting that many work reorganization initiatives focused on rationalization activities may be counterproductive and damaging, causing feelings of alienation and disillusionment among frontline staff and resulting in increased stress, prolonged feelings of anxiety, and depressive symptoms [43,44].

In confirming hypotheses 2 and 3, this research also provides new insights to improve our understanding of organizational change. This study demonstrated that an increase in burnout over time influenced employees’ attitudes toward change, presumably through their adoption of more conservative strategies for investing their available resources. A negative association was found between increase in burnout over time and commitment to change. A positive association was found between increase in burnout over time and cynicism toward change management. Moreover, the increase in burnout seemed to completely mediate the relationships between the majority of decreases of resources over time and attitudes toward change. These results support the contention that when employees experience an increase in burnout associated with a perceived loss of resources, they may become more cautious in investing their remaining resources. Specifically, our findings suggest that individuals with emotional exhaustion tend to take steps to protect their remaining resources, instead of investing them into a change project. Our results also echo certain theories on social influence, policy implementation, and innovation diffusion that suggest an agent will either cooperate with or resist organizational change after considering the gains and losses involved or doing a rational analysis of benefits and costs [45,46,47]. As such, attitudinal and behavioural support to change may be undermined by conditions that threaten employees and affect their emotional state and psychological well-being. Note that a partial mediation was found for group cohesion, suggesting that other mechanisms might be at play to explain the relationship between loss of group cohesion and negative attitudes toward organizational change. Future research might explore how group resources differ from personal resources in the attitudes development process.

Some scholars have argued that studies on organizational change have often focused on the macro level, restricting attention to issues such as culture, communication, training, and technology, while neglecting micro level issues related to individuals [48,49]. This research shows that the emotional states and subsequent behaviours and attitudes that result from individuals’ perceptions about a proposed change are brought into play during an organizational change. The dynamic and complex relationships revealed in this study between perceptions of loss of resources, burnout, and attitudes to change indicate that the difficulties often encountered in implementing changes do not come simply from obstructionist behaviours by agents inherently resistant to change. Rather, the attitudes and actions of agents resisting change are shaped by the interplay of a series of factors and disruptions that may be associated with the change process. Such results call into question many current organizational change processes in healthcare and highlight the importance of developing strategies that do not marginalize or alienate frontline staff. While change failures are often occasions for blame games, our results clearly divert the locus of blame away from individuals by revealing a complex set of factors operating at different levels (task, supervision, interpersonal, organizational) and influencing individual perceptions and actions. On a practical level, if healthcare organizations want to promote attitudes that foster change, they should invest in a broad set of interventions that are not restricted to demand-reducing strategies but also include resource-development strategies. For example, it could be that an increase in supervisor support over time plays an important role in buffering the detrimental effects of the other decreases in resources we found. Future research with larger samples could make it possible to develop moderated-mediated models to test this specific hypothesis.

This study’s findings should be interpreted in light of at least three limitations. First, this study was based on survey data and relied solely on self-report measures, which are prone to many kinds of response bias and common method variance [50]. Common method variance may have artificially inflated associations between the constructs studied through factors such as social desirability [51]. These problems could be minimized in future research by using multisource data and combining other measures, such as supervisor ratings of job resources and attitude to change, with self-report inventories.

Second, there are specificities in our sample related to gender and profession that warrant discussion. The nursing profession is female-dominated, and in this study, 87.5% of respondents were women. Future research should examine whether individual differences, specifically gender sensitivity to resource loss and gender difference in value attached to some resources, influence burnout and attitude to change. In addition, the impact of an organizational change on a group such as nurses may be different in many respects from the impact on other professionals. Third, our study shares the limitations inherent in real-world intervention studies with regard to uncovering unequivocal cause-and-effect relationships. Although the use of a longitudinal design and an LDS analytical approach might have prevented some biases, we cannot entirely preclude the possibility that the presence of residual confounders may explain, at least in part, the associations revealed. 

Despite these limitations, the findings of this research provide considerable support to the application of the conservation of resources theory to the domain of organizational change. The study’s main merit was in the use of a two-wave longitudinal design, which enabled us to detect relationships between differences in job resources and burnout, and the subsequent impact on attitudes to change. While burnout is an end result whose absolute level may be lower than other intermediate results such as stress, our findings are even more important as they reveal not only significant variations in burnout across time but also significant relationships between those variations and perceived loss of resources. In future research, a three-wave longitudinal design may provide additional insights by depicting the burnout process more precisely, especially between two adjacent periods (T1 to T2 and T2 to T3), or modelling the trajectory (e.g., linear, curvilinear) over time (T1 to T3) for the different variables studied. 
